# Dairy product consumption and type 2 diabetes among Korean adults: a prospective cohort study based on the Health Examinees (HEXA) study

**DOI:** 10.4178/epih.e2022019

**Published:** 2022-02-04

**Authors:** Jiaqi Zhang, Kyungjoon Lim, Sangah Shin

**Affiliations:** 1 Department of Food and Nutrition, Chung-Ang University, Anseong, Korea; 2 Faculty of Medicine and Health, School of Medical Science, University of Sydney, Melbourne, Australia

**Keywords:** Dairy products, Yogurt, Type 2 diabetes, Cohort studies, Adults

## Abstract

**OBJECTIVES:**

It has been suggested that the consumption of dairy products helps lower the prevalence of type 2 diabetes (T2D). We investigated the association between the consumption of dairy products and T2D events in middle-aged Korean adults.

**METHODS:**

We followed up 53,288 participants (16,895 male and 36,393 female) in the Health Examinees (HEXA) study. The consumption of dairy products was assessed using the self-administered food frequency questionnaire, and T2D was defined according to the 2015 treatment guidelines of the Korean Diabetes Association. Hazard ratios (HRs) and 95% confidence intervals (CIs) between the consumption of dairy products and the risk of T2D were calculated using Cox proportional hazards models after adjusting for potential confounders. Spline regression was used to better represent the association between the consumption of dairy products and the risk of T2D.

**RESULTS:**

Among male, those with higher consumption of dairy products had a significantly lower risk of T2D than those who consumed essentially no dairy products (HR, 0.73; 95% CI, 0.58 to 0.91). In particular, consumption of yogurt (HR, 0.75; 95% CI, 0.60 to 0.93; p_trend_=0.035) and cheese (HR, 0.66; 95% CI, 0.49 to 0.89; p_trend_=0.005) was negatively associated with the incidence of T2D in male. In female, daily consumption of 1 serving of yogurt decreased the risk of T2D by 11%.

**CONCLUSIONS:**

The association between the consumption of dairy products and the risk of T2D differed by sex and dairy product type. Further prospective studies are needed to confirm these associations.

## GRAPHICAL ABSTRACT

[Fig f3-epih-44-e2022019]

## INTRODUCTION

Type 2 diabetes (T2D) is among the most prevalent chronic diseases worldwide, with 9.3% of adults (around 463 million) living with the condition [1], and its incidence is increasing. The International Diabetes Federation projects that approximately 578 million adults will have T2D by 2030 [2]. Diabetes is a disease of concern in Korea; in 2020, T2D was prevalent in 13.8% of adults (approximately 4.94 million) aged over 30 years [3]. T2D is a serious chronic disease caused by a complex combination of genetic and environmental interaction and other risk factors such as lifestyle [4]. Thus, it is important to understand the modifiable factors associated with the risk of T2D.

One of these modifiable risk factors is diet, in particular, dairy products. Dairy products contain saturated fat, which is generally considered to have adverse effects on health [5]. Excessive intake of saturated fat increases the risk of cardiovascular disease [6]. Regular consumption of milk protein causes the insulin-like growth factor-1 (IGF-1) axis to permanently increase serum IGF-1 levels. Insulin/IGF-1 signaling is closely linked to the development of chronic diseases such as T2D, obesity, and cancer [7]. However, there is also increasing evidence supporting a possible benefit of dairy products in the management of T2D. Dairy products contain calcium, vitamin D, whey protein, and flavonoids, which have a favorable effect on glucose homeostasis and thus improve T2D [8,9]. In addition, the lactic acid bacteria found in fermented dairy products, such as yogurt and cheese, contribute to intestinal microbial balance [10], which may improve T2D by reducing the risk of obesity [11].

In several studies, dairy products were divided into milk, yogurt, and cheese [12-14]. A large cohort study of Chinese adults found that daily milk intake was associated with a significant reduction in the risk of T2D [15]. A study on yogurt intake and the risk of diabetes, which evaluated 3 cohorts of the United States adults, found that increased yogurt intake was associated with a reduced risk of T2D [16]. In the European Prospective Investigation into Cancer and Nutrition study, a negative association between diabetes and cheese intake was found [17]. However, most of these studies were focused on the association between dairy product intake and the risk of T2D in Western countries. Furthermore, most current studies on dairy consumption and T2D in Korea are cross-sectional studies [18] with inadequate data [19]. The variety of dairy products and dairy product intake are lower in Korea than in Western countries [12,13,20]. Therefore, cohort studies on the association between the consumption of dairy products and the risk of T2D in Korea are limited.

Given the increasing incidence of T2D among Koreans [3], it is crucial to understand the significance of the consumption of dairy products for the prevention of T2D. Thus, this study aimed to investigate the effect of consumption of dairy products, including milk, yogurt, and cheese, on the incidence of T2D among Korean adults aged 40-69 years, using data from a large Korean population.

## MATERIALS AND METHODS

### Study population

The Health Examinees (HEXA) study was a part of the Korean Genome and Epidemiology Study, a large, prospective, community-based genomic cohort study. The HEXA study was conducted to investigate the cause of major chronic diseases in Koreans [21]. The baseline survey for the HEXA study was conducted from 2004 to 2013 at 38 hospitals and local health screening centers following strict standardized study procedures. In total, 65,642 participants completed the initial and follow-up surveys between 2012 and 2016 [21].

**Figure 1.**
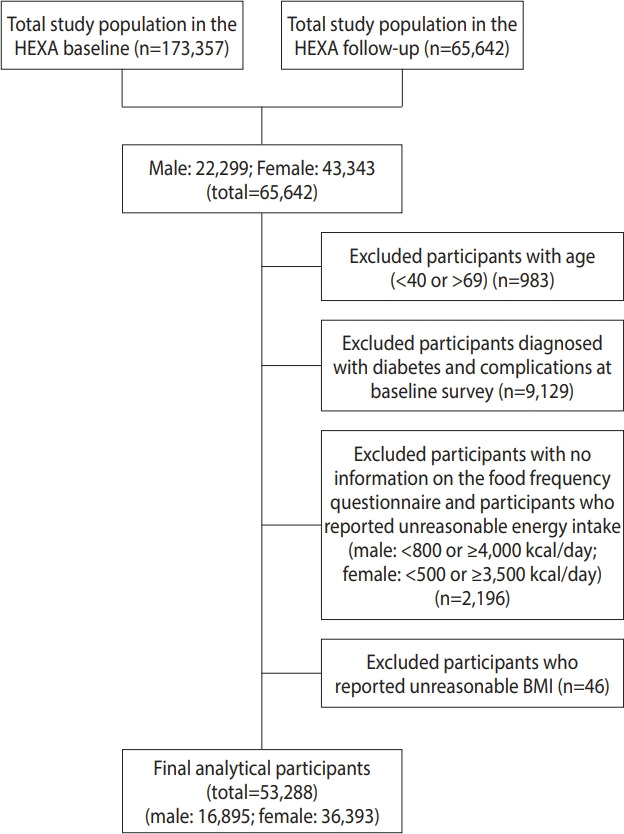
Participant inclusion flowchart. HEXA, Health Examinees; BMI, body mass index.

Among the 65,642 participants who completed the baseline and follow-up surveys, we excluded those aged < 40 years and > 69 years (n = 983) and those who were diagnosed with T2D and complications at the baseline survey (n = 9,129). Further, we excluded those without dietary intake records or those who reported an unreasonable amount of energy intake ( < 800 or ≥ 4,000 kcal/day in male and < 500 or ≥ 3,500 kcal/day in female; n= 2,196). Participants with missing information on body mass index (BMI; n= 46) were also excluded. Finally, 53,288 participants (16,895 male and 36,393 female) were included in the final analysis (Figure 1).

### Assessment of dairy product consumption

Food intake in the past year was assessed using a 106-item selfadministered food frequency questionnaire (FFQ). The frequency of food consumption was classified into 9 categories: never or almost never, once a month, 2 to 3 times a month, once or twice a week, 3 to 4 times a week, 5 to 6 times a week, once a day, twice a day, and 3 times a day. The portion sizes were categorized into 3 groups: half standard serving, 1 standard serving, and 2 standard servings. The dairy products in the FFQ included 3 types: milk, yogurt, and cheese. One serving size was equal to 200 mL for milk, 120 mL for yogurt, and 20 g for cheese. The consumption of dairy products was converted to a weekly frequency and multiplied by the number of servings reported for each food. Based on their reported distribution of each target dairy product, the participants were categorized into 3 groups for consumption of cheese (none, < 1, and ≥ 1/wk) and 4 groups for consumption of dairy products, milk, and yogurt (none, ≤ 2, > 2-< 7/wk, and ≥ 1/day).

To verify the validity and 1-year repeatability of the FFQ, information was collected in each of the 4 seasons of the 1-year period using the 3-day diet record method. The correlation coefficients between the FFQ and 12-day diet record, adjusted for attenuation, age, sex, and energy intake in the Korean population, ranged between 0.23 and 0.64, with 0.45 being the value for nutrient intake and 0.39 for nutrient density [22].

### Definition of type 2 diabetes

T2D was defined according to the 2015 treatment guidelines of the Korean Diabetes Association as a fasting blood glucose level ≥ 126 mg/dL or active treatment with glucose-lowering medication (insulin or oral hypoglycemic agents), at a follow-up survey [23]. The number of person-years was calculated by multiplying the average number of participants by the number of years of observation.

### Covariates

Covariates included socio-demographic factors such as age, BMI, educational level, and health behavior. Education levels were divided into 3 categories: middle school or less, high school or college, and undergraduate school or higher. Smoking status was assessed using the question “Have you smoked 5 packs of cigarettes (100 cigarettes) in your lifetime?” Participants who answered “no” were defined as non-smokers. Meanwhile, those who answered “yes” were categorized as current smokers or ever-smokers if they still smoked or no longer smoked at the time of the survey, respectively. Drinking status was determined by the question “Are you unable to drink alcohol or do you choose not to drink alcohol?” Participants who answered “no” were identified as non-drinkers, while those who answered “yes” were classified as current drinkers. Physical activity was assessed with the question “Do you regularly participate in any sport?” Participants who answered “yes” were assigned to the regular exercise group, while those who answered “no” were assigned to the non-regular exercise group.

### Statistical analysis

All analyses to investigate the association between the consumption of dairy products and the risk of T2D were conducted separately by sex. Categorical variables were analyzed using the chisquare test, while continuous variables were analyzed using general linear regression. The association between consumption of dairy products and the development of T2D was determined by calculating hazard ratios (HRs) and 95% confidence intervals (CIs) using Cox proportional hazard models. We used multivariate models adjusted for age, BMI, educational level (≤ middle school, high school or college, undergraduate school or higher), smoking status (never, ever, current, unknown), alcohol drinking status (never, ever, unknown), physical activity (yes, no, unknown), and total energy intake as covariates. To test for a linear trend between the consumption of dairy products and HR for T2D, participants were assigned a median value for each category, and this variable was entered into the model as a continuous term. The coefficient was assessed using the Wald test. To assess the linearity of the association between the consumption of dairy products and the risk of T2D, we used spline regression analysis. Constrained cubic splines were fitted to logistic and proportional risk regression models to non-parametrically examine the relationship between the consumption of dairy products and the HR for T2D, with 4 knots at fixed percentiles (5th, 35th, 65th, and 95th percentiles). We used the restricted maximum likelihood method in our analysis, combining the 2 regression coefficients and variance matrices estimated in this study. The pooled relative risk for a given exposure value was then estimated. The p-values for non-linearity were calculated by testing the null hypothesis that the coefficients of the second spline were equal to 0. All statistical analyses were performed using SAS version 9.4 (SAS Institute Inc., Cary, NC, USA). A p-value ≤ 0.05 was considered to indicate statistical significance.

### Ethics statement

All participants voluntarily signed an informed written consent form prior to enrollment. The current study was performed in accordance with the guidelines specified in the declaration of Helsinki, and the study protocol was approved by the local Institutional Review Board (IRB) of the Ethics Committee of the Korean Genome and Epidemiology Study of the Korea National Institute of Health (IRB No. 2014-08-02-3C_A).

## RESULTS

The characteristics of the participants according to the consumption of dairy products are shown in Table 1. Compared with male who consumed no or less dairy products every week, those who consumed dairy products once or more per day were younger, with higher education levels, and were more likely to perform regular exercise. Similarly, female who consumed dairy products at least once a day were younger, had higher education levels, were less likely to be smokers, and were regular exercisers. Both male and female with a higher consumption of dairy products also consumed higher amounts of energy, carbohydrates, protein, and fat. The trends in the characteristics of the participants according to their consumption of milk, yogurt, and cheese were similar to those according to the total consumption of dairy products (Supplementary Materials 1-3).

Table 2 shows the multivariate HRs (95% CI) for T2D according to the consumption of total dairy products, milk, yogurt, and cheese. Among male, those who consumed dairy products, including yogurt and cheese, had a lower risk of T2D than those who did not consume dairy products. In the comparison between the highest and lowest (non)-intake categories, the multivariate HRs (95% CIs) for the consumption of total dairy products, yogurt, and cheese were 0.73 (95% CI, 0.58 to 0.91), 0.75 (95% CI, 0.60 to 0.93), and 0.69 (95% CI, 0.51 to 0.94), respectively. However, in dairy products, the p for trend was not significant. In female, there was no significant association for the consumption of total dairy products, milk, yogurt, and cheese. However, increasing the consumption of yogurt by 1 serving/day may reduce the risk of T2D by 11% (HR, 0.89; 95% CI, 0.79 to 0.99).

**Figure 2.**
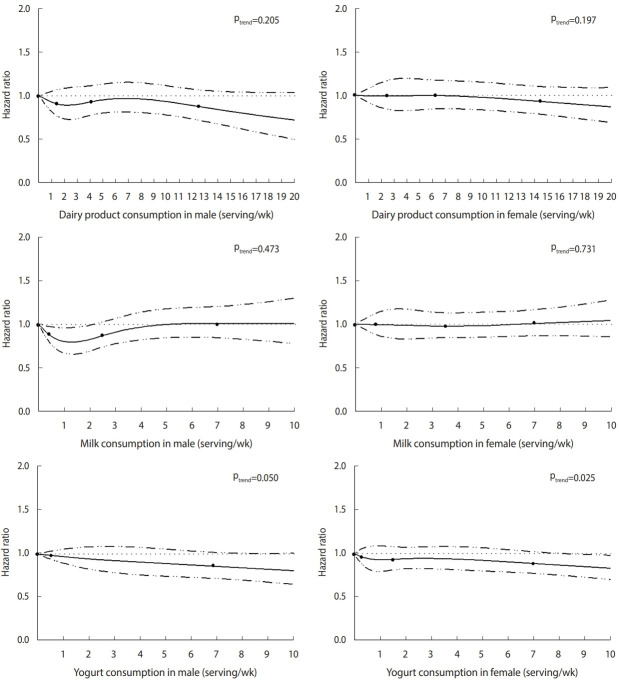
Hazard ratios of dairy product consumption for type 2 diabetes, calculated using spline regression. Hazard ratios are shown as solid lines, and 95% confidence intervals are shown as dashed lines. The 4 knots of dairy products for male: 0.00, 1.44, 4.29, and 12.48. The 4 knots of dairy products for female: 0.00, 2.54, 6.29, and 14.58. The 4 knots of milk for male: 0.00, 0.40, 2.50, and 7.00. The 4 knots of milk for female: 0.00, 0.86, 3.50, and 7.00. The 3 knots of yogurt for male: 0.00, 0.58, and 7.00. The 4 knots of yogurt for female: 0.00, 023, 1.50, and 7.00. The models are adjusted for age, body mass index, educational level, smoking status, alcohol drinking status, and physical activity.

Spline regression was used to better characterize the association between the consumption of dairy products and the risk of T2D. As shown in Figure 2, the 4 knots of the consumption of dairy products for male were 0.00, 1.44, 4.29, and 12.48, while those for female were 0.00, 2.54, 6.29, and 14.58. The 4 knots of the consumption of milk for male were 0.00, 0.40, 2.50, and 7.00, while those for female were 0.00, 0.86, 3.50, and 7.00. We divided the male in the yogurt intake category into 3 knots (0.00, 0.58, and 7.00) because of the uneven distribution of yogurt intake. The 4 knots of the consumption of yogurt for female were 0.00, 0.23, 1.50, and 7.00. The models were adjusted for age, BMI, education level, smoking status, alcohol consumption status and physical activity, and we found that the consumption of dairy products and milk had no correlation with the risk of T2D. However, there was a linear inverse association between yogurt intake and the incidence of T2D in both male (p= 0.050) and female (p= 0.025).

## DISCUSSION

The association between the consumption of different types of dairy products and the risk of T2D remains controversial. This study found that consumption of dairy products was associated with a lower risk of T2D among male. In particular, the consumption of yogurt and cheese was inversely associated with the incidence of T2D in male. In female, increasing the daily consumption by 1 serving of yogurt decreased the risk of T2D by 11%.

Our findings regarding the association between the consumption of dairy products and the risk of T2D are consistent with those of other studies [5,16,24,25]. A dose-response meta-analysis of observational studies showed similar results to those of our study. That study combined data from 22 prospective cohort studies and found that the consumption of yogurt and dairy products showed significant non-linear inverse associations with the risk of T2D, while no significant association was observed for milk [26]. A meta-analysis of 14 prospective cohort studies showed that higher yogurt intake was associated with a reduced risk of T2D [16]. In a systematic evaluation and meta-analysis, cheese and yogurt intake showed significant negative associations with the risk of T2D, while no association was found between whole milk intake and the risk of T2D [10].

However, a recent study of 393 T2D patients from Rotterdam, the Netherlands, found no association between the consumption of dairy products and the risk of T2D [27]. Similarly, the European Prospective Investigation into Cancer and Nutrition study found no association between the total consumption of dairy products and the risk of T2D [17]. These conflicting findings may be due to differences in the definition of T2D, the geographical distribution of study populations, and the analysis methods. In the current study, the consumption of dairy products, particularly yogurt, was associated with a reduced risk of T2D in Korean adults. This finding may be related to the dairy intake pattern of Koreans. Due to lactose intolerance, many Koreans prefer to consume yogurt, which is better for intestinal health than milk [28]. There were also significantly more people who consumed at least 1 serving/day of yogurt than those who consumed at least 1 serving/day of milk. In a narrative review of the latest evidence on the benefit of consumption of dairy products for the prevention of T2D, daily intake of 3 servings of dairy products, particularly yogurt and cheese, lowered the risk of T2D [29]. A cross-sectional study on the association between dairy subgroups and prediabetes and newly diagnosed T2D among Dutch adults found a negative association between the consumption of skim and fermented dairy products and the risk of T2D [30].

Although cheese is a fermented food like yogurt, the majority of participants in this study consumed less than 1 serving of cheese per week. This could explain the finding that cheese had a weaker effect than yogurt. Nevertheless, cheese intake was found to be beneficial for preventing T2D in male. Cheese might be a good source of vitamin K2, which is responsible for protein carboxylation by osteocalcin [10]. Osteocalcin concentration may affect insulin sensitivity and T2D by regulating the expression of insulin genes and β-cell proliferation markers [31]. In the current study, the consumption of dairy products was higher in male than in female. This could be because Korean male are more concerned about their health than Korean female [32,33].

Certain components of dairy products, such as calcium, vitamin D, whey protein, and magnesium, have been suggested to be beneficial for T2D [34]. These components can positively affect glucose homeostasis [8]. The most active form of vitamin D is 1,25(OH)_2_D_3_ [9]. Calcium^2+^ signaling induced by 1,25(OH)_2_D_3_ regulates insulin secretion from pancreatic β-cells [35]. Prospective studies have reported that calcium intake lowers the prevalence of T2D [36]. Whey protein may also improve T2D, as it is composed of glycopeptides, β-lactoglobulin, α-lactalbumin, and lactoferrin, and it improves glucose clearance through differential upregulation of glucoregulatory transcripts in the liver and skeletal muscle [37]. However, in our study, those who consumed dairy products had a lower risk of T2D than those who did not consume dairy products, but the p trend values were showing no significance. This may be because the HR values had a trend of rising and falling.

Several mechanisms could explain the negative association between yogurt intake and the risk of T2D. Obesity is one of the main causes of T2D [38]. The probiotics in yogurt reduce the risk of obesity, and this in turn may reduce the risk of T2D [11,39]. Further, yogurt intake can improve glucose homeostasis and glucose metabolism by regulating hepatic gluconeogenesis [40]. Oxidative stress plays a major role in T2D [41], and probiotics in yogurt have antioxidative mechanisms, including scavenging of reactive oxygen species, inhibiting enzymes, and lowering the activity of lactic acid bacteria [42]. The *Lactobacillus* fermentum ME-3 increases the total antioxidant status and decreases oxidative stress indicators [43]. *Lactobacillus acidophilus* and *Lactobacillus* have been shown to reduce oxidative stress and have anti-T2D effects [44].

This study had some limitations. First, information on the consumption of dairy products was obtained from a self-reported quantitative questionnaire, and thus, the possibility of measurement error in the dietary assessment cannot be excluded. Second, most Korean adults consume whole milk; as such, we were unable to obtain information from the FFQ on the effect of milk by type (whole, low-fat, and skim) on the risk of T2D. Finally, the time of T2D onset could not be accurately determined due to the limited follow-up period. Although we carefully adjusted for relevant confounders, some unmeasured and residual confounders related to the consumption of dairy products and T2D may still have been presented.

Despite these limitations, our study has many strengths. The main advantage is that, to our best knowledge, this is the first large-scale prospective cohort study to evaluate the impact of the consumption of dairy products on the risk of T2D in Korea. Given that this study considered the pattern of consumption of dairy products among Koreans, the data can be used as evidence for establishing guidelines for the consumption of dairy products in Korea. In addition, we adjusted for potential key confounders to better analyze the independent association between the consumption of dairy products and the risk of T2D. More prospective studies are required to further determine the potential benefits of consumption of dairy products in the management of T2D.

In conclusion, the consumption of 2 or more servings of yogurt per week reduced the incidence of T2D among Korean adults compared with no or rare consumption of yogurt. In contrast, no significant association was found between the consumption of milk and the risk of T2D. Therefore, further prospective studies and clinical trials are needed to test these findings.

## Figures and Tables

**Figure f3-epih-44-e2022019:**
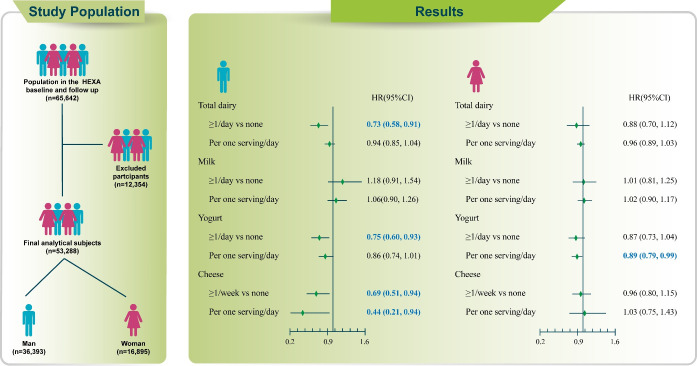


**Table 1.** Characteristics of participants from the HEXA study by total dairy product consumption

Characteristics			Dairy product consumption (serving)				p-value^1^
			Non	≤2/wk	>2-<7/wk	≥1/day	
Male (n=16,895)			1,700	5,537	5,746	3,912	
	Person-years		8,220.4	27,426.1	28,523.3	19,086.4	
	Cases/total participants (n)		136/1,700	323/5,537	364/5,746	222/3,912	
	Age (yr)		60.2±8.1	59.0±8.2	58.5±8.4	59.4±8.4	<0.001
	BMI (kg/m^2^)		24.2±2.8	24.4±2.7	24.3±2.7	24.3±2.7	0.047
	Educational level						<0.001
		Middle school or less	503 (29.9)	1,257 (23.0)	1,012 (17.8)	641 (16.6)	
		High school or college	684 (40.7)	2,248 (41.1)	2,322 (40.8)	1,510 (39.0)	
		Undergraduate school or higher	494 (29.4)	1,967 (36.0)	2,356 (41.4)	1,718 (44.4)	
	Smoking						<0.001
		Non-smokers	409 (24.1)	1,603 (29.0)	1,873 (32.7)	1,365 (35.0)	
		Ever smokers	698 (41.2)	2,290 (41.5)	2,346 (41.0)	1,624 (41.7)	
		Current smokers	589 (34.7)	1,629 (29.5)	1,501 (26.2)	909 (23.3)	
	Alcohol drinking						0.007
		Non-drinkers	322 (20.3)	1,085 (20.9)	1,117 (20.8)	852 (23.5)	
		Current drinkers	1,266 (79.7)	4,098 (79.1)	4,253 (79.2)	2,779 (76.5)	
	Regular exercise						<0.001
		No	816 (48.2)	2,464 (44.6)	2,235 (39.1)	1,311 (33.6)	
		Yes	878 (51.8)	3,059 (55.4)	3,488 (61.0)	2,590 (66.4)	
	Dietary intake						
		Total energy intake (kcal/day)^2^	1,585.4±378.1	1,651.9±403.5	1,756.9±434.4	1,850.6±488.0	<0.001
		Protein (g/day)	54.8±20.5	56.0±19.9	64.6±21.9	72.8±25.4	<0.001
		Protein (%)	13.0±2.6	13.0±2.3	13.6±2.3	14.1±2.4	<0.001
		Fat (g/day)	24.1±14.5	25.5±13.9	31.9±15.3	37.2±17.9	<0.001
		Fat (%)	12.6±5.3	13.0±4.9	14.9±4.8	16.0±5.0	<0.001
		Carbohydrate (g/day)	306.3±74.8	313.5±72.9	333.2±79.2	354.5±87.2	<0.001
		Carbohydrate (%)	74.3±7.5	74.0±6.8	71.4±6.6	69.9±6.8	<0.001
Female (n=36,393)			2,056	8,751	13,768	11,818	
	Person-years		9,977.1	43,661.2	69,322.1	59,393.6	
	Cases/total participants (n)		88/2,056	331/8,751	496/13,768	420/11,818	
	Age (yr)		58.7±8.0	57.1±7.8	56.8±7.5	57.8±7.3	<0.001
	BMI (kg/m^2^)		23.8±3.1	23.7±3.1	23.6±2.9	23.4±2.9	<0.001
	Educational level						<0.001
		Middle school or less	958 (47.2)	3,359 (38.8)	4,533 (33.3)	3,555 (30.5)	
		High school or college	811 (39.9)	3,794 (43.8)	6,215 (45.7)	5,387 (46.2)	
		Undergraduate school or higher	263 (12.9)	1,507 (17.4)	2,866 (21.1)	2,728 (23.4)	
	Smoking						0.004
		Non-smokers	1,984 (96.8)	8,472 (97.3)	13,378 (97.7)	11,479 (97.5)	
		Ever-smokers	21 (1.0)	71 (0.8)	113 (0.8)	129 (1.1)	
		Current smokers	44 (2.2)	161 (1.9)	205 (1.5)	160 (1.4)	
	Alcohol drinking						<0.001
		Non-drinkers	1,495 (74.1)	6,043 (70.4)	8,999 (67.0)	7,980 (68.9)	
		Current drinkers	524 (26.0)	2,538 (29.6)	4,442 (33.1)	3,611 (31.2)	
	Regular exercise						<0.001
		No	1,171 (57.1)	4,591 (52.6)	6,433 (46.9)	4,690 (39.8)	
		Yes	881 (42.9)	4,137 (47.4)	7,285 (53.1)	7,091 (60.2)	
	Dietary intake						
		Total energy intake (kcal/day)^2^	1,411.4±384.7	1,450.0±412.9	1,539.1±436.0	1,621.3±470.5	<0.001
		Protein (g/day)	47.2±17.2	49.1±18.3	56.2±19.8	65.5±23.0	<0.001
		Protein (%)	12.7±2.4	12.9±2.4	13.5±2.4	14.2±2.5	<0.001
		Fat (g/day)	18.1±10.7	20.4±12.0	25.9±13.3	32.0±15.3	<0.001
		Fat (%)	10.8±4.8	11.8±4.9	13.9±4.9	15.4±5.1	<0.001
		Carbohydrate (g/day)	280.0±74.4	285.0±78.7	299.5±82.9	322.8±88.3	<0.001
		Carbohydrate (%)	76.5±6.7	75.4±6.8	72.6±6.8	70.4±7.0	<0.001

Values are presented as the mean±SD or as number (%); Continuous variables are reported as the mean±SD, while categorical variables are reported as number (%). HEXA, Health Examinees; BMI, body mass index; SD, standard deviation.

1 Categorical and continuous variables were calculated using the chi-square test and general linear regression, respectively.

2 Total energy intake was adjusted using the residual method.

**Table 2.** HRs and 95% CIs of type 2 diabetes according to dairy product consumption in the HEXA study population

Variables			Dairy product consumption (servings)				p_trend_^1^	HR for each additional serving/day
			None	≤2/wk	>2-<7/wk	≥1/day		
Male (n=16,895)								
	Total dairy product consumption							
		Cases/total participants (n)	136/1,700	323/5,537	364/5,746	222/3,912		
		Person-year	8,220.4	27,426.1	28,523.3	19,086.4		
		Model 1	1.00 (reference)	0.66 (0.54, 0.80)	0.70 (0.57, 0.85)	0.65 (0.53, 0.81)	0.101	0.90 (0.81, 0.99)
		Model 2	1.00 (reference)	0.66 (0.54, 0.82)	0.74 (0.60, 0.90)	0.73 (0.58, 0.91)	0.694	0.94 (0.85, 1.04)
	Milk consumption							
		Cases/total participants (n)	219/3,318	439/7,152	304/5,087	83/1,338		
		Person-years	16,042.3	35,867.4	25,108.9	6,237.6		
		Model 1	1.00	0.81 (0.69, 0.96)	0.82 (0.69, 0.98)	1.05 (0.81, 1.35)	0.709	1.00 (0.85, 1.18)
		Model 2	1.00	0.81 (0.68, 0.95)	0.84 (0.70, 1.00)	1.18 (0.91, 1.54)	0.194	1.06 (0.90, 1.26)
	Yogurt consumption							
		Cases/total participants (n)	422/6,276	404/6,826	114/1,882	105/1,911		
		Person-years	30,670.6	33,586.3	9,375.6	9,623.7		
		Model 1	1.00 (reference)	0.84 (0.74, 0.97)	0.83 (0.67, 1.02)	0.71 (0.57, 0.88)	0.005	0.82 (0.70, 0.95)
		Model 2	1.00 (reference)	0.85 (0.74, 0.98)	0.90 (0.72, 1.11)	0.75 (0.60, 0.93)	0.035	0.86 (0.74, 1.01)
			**Non**	**<1/wk**	**≥1/wk**			
	Cheese consumption							
		Cases/total participants (n)	715/10,850	280/4,941	50/1,104			
		Person-year	53,631.6	24,167.2	5,457.4			
		Model 1	1.00 (reference)	0.86 (0.75, 0.99)	0.66 (0.49, 0.88)		0.003	0.37 (0.18, 0.77)
		Model 2	1.00 (reference)	0.89 (0.76, 1.03)	0.69 (0.51, 0.94)		0.016	0.44 (0.21, 0.94)
Female (n=36,393)								
	Total dairy product consumption							
		Cases/total participants (n)	88/2,056	331/8,751	496/13,768	420/11,818		
		Person-years	9,977.1	43,661.2	69,322.1	59,393.6		
		Model 1	1.00 (reference)	0.83 (0.65, 1.04)	0.79 (0.63, 0.99)	0.76 (0.60, 0.96)	0.085	0.91 (0.85, 0.98)
		Model 2	1.00 (reference)	0.85 (0.67, 1.08)	0.87 (0.69, 1.10)	0.88 (0.70, 1.12)	0.987	0.96 (0.89, 1.03)
	Milk consumption							
		Cases/total participants (n)	201/5,277	495/13,011	489/13,900	150/4,205		
		Person-years	25,722.4	66,000.1	69,972.6	20658.9		
		Model 1	1.00 (reference)	0.91 (0.77, 1.08)	0.87 (0.74, 1.03)	0.92 (0.74, 1.13)	0.387	0.95 (0.84, 1.09)
		Model 2	1.00 (reference)	0.93 (0.78, 1.09)	0.91 (0.77, 1.08)	1.01 (0.81, 1.25)	0.871	1.02 (0.90, 1.17)
	Yogurt consumption							
		Cases/total participants (n)	415/10,564	549/15,143	183/5,344	188/5,342		
		Person-years	51,965.4	75,878.0	27,352.3	27158.3		
		Model 1	1.00 (reference)	0.91 (0.80, 1.04)	0.79 (0.67, 0.94)	0.81 (0.68, 0.96)	0.008	0.85 (0.76, 0.95)
		Model 2	1.00 (reference)	0.94 (0.83, 1.08)	0.88 (0.74, 1.05)	0.87 (0.73, 1.04)	0.115	0.89 (0.79, 0.99)
			**Non**	**<1/wk**	**≥1/wk**			
	Cheese consumption							
		Cases/total participants (n)	787/19,305	397/12,182	151/4,906			
		Person-years	97,340.2	60,771.0	24,242.8			
		Model 1	1.00 (reference)	0.88 (0.78, 0.99)	0.84 (0.70, 1.00)		0.064	0.85 (0.61, 1.18)
		Model 2	1.00 (reference)	0.94 (0.83, 1.07)	0.96 (0.80, 1.15)		0.584	1.03 (0.75, 1.43)

Model 1 is adjusted for age (continuous); Model 2 is adjusted for age (continuous), body mass index (continuous), educational level (≤middle school, high school or college, ≥undergraduate school), smoking status (never, ever, current, unknown), alcohol drinking status (never, ever, unknown), physical activity (yes, no, unknown), and total intake (continuous). HR, lhazard ratio; CI, confidence interval; HEXA, Health Examinees.

1 Calculated by categories of milk consumption; Associations were tested using the median values for each category and were treated as continuous variables.

## SUPPLEMENTARY MATERIALS

Supplementary Material 1. Participant characteristics by milk consumption by the HEXA study population

Supplementary Material 2. Participant characteristics by yogurt consumption by the HEXA study population

Supplementary Material 3. Participant characteristics by cheese consumption by the HEXA study population
