# Supplementary material for: Dairy product consumption and type 2 diabetes among Korean adults: a prospective cohort study based on the Health Examinees (HEXA) study
Source: Epidemiol Health. 2022 Feb 4;44:e2022019. doi: 10.4178/epih.e2022019 (PMC9117095; doi:10.4178/epih.e2022019)
Supplement: Supplementary Material 1. — Participant characteristics by milk consumption by the HEXA study population [file epih-44-e2022019-suppl1.docx]

Supplementary Material 1. Participant characteristics by milk consumption by the HEXA study population

| Milk consumption (Serving) | | | | | |
| --- | --- | --- | --- | --- | --- |
| Variables^1^ | Non | ≤2/week | >2-<7/week | ≥1/day | *p*-Value^2^ |
| Men (n=16 895) | 3 318 | 7 152 | 5 087 | 1 338 |  |
| Person-year | 16 042.3 | 35 867.4 | 25 108.9 | 6 237.6 |  |
| Cases/Total participants (n) | 219/3 318 | 439/7 152 | 304/5 087 | 83/1 338 |  |
| Age (years) | 59.9 ± 8.2 | 58.8 ± 8.3 | 58.7 ± 8.4 | 59.4 ± 8.4 | <0.0001 |
| BMI (kg/m^2^) | 24.1 ± 2.7 | 24.4 ± 2.7 | 24.3 ± 2.7 | 24.4 ± 2.8 | 0.0004 |
| Educational level |  |  |  |  | <0.0001 |
| Middle school or less | 808 (24.7) | 1 502 (21.2) | 864 (17.2) | 239 (18.0) |  |
| High school or college | 1 335 (40.7) | 2 829 (40.0) | 2 094 (41.6) | 506 (38.1) |  |
| Undergraduate school or higher | 1 134 (34.6) | 274 5(38.8) | 2 074 (41.2) | 582 (43.9) |  |
| Smoking, n (%) |  |  |  |  | <0.0001 |
| Non | 889 (26.9) | 2 222 (31.2) | 1 670 (33.0) | 469 (35.3) |  |
| Ever | 1 357 (41.0) | 2 914 (40.9) | 2 135 (42.1) | 552 (41.5) |  |
| Current | 1 065 (32.2) | 1 991 (27.9) | 1 263 (24.9) | 309 (23.2) |  |
| Alcohol drinking, n (%) |  |  |  |  | 0.1143 |
| Non | 671 (21.7) | 1 381 (20.6) | 1 034 (21.9) | 290 (23.3) |  |
| Current | 2 429 (78.4) | 5 319 (79.4) | 3 695 (78.1) | 953 (76.7) |  |
| Regular exercise |  |  |  |  | <0.0001 |
| No | 1 480 (44.7) | 3 027 (42.5) | 1 869 (36.9) | 450 (33.7) |  |
| Yes | 1 829 (55.3) | 4 102 (57.5) | 3 200 (63.1) | 884 (66.3) |  |
| Dietary intake |  |  |  |  |  |
| Total energy intake (kcal/day) | 1 618.8±392.5 | 1 687.0±416.6 | 1 797.0±454.6 | 1 941.7±511.0 | <0.0001 |
| Protein (g/day) | 57.0±21.1 | 59.4±21.5 | 67.5±23.5 | 75.6±25.4 | <0.0001 |
| Protein (%) | 13.1±2.5 | 13.2±2.3 | 13.8±2.3 | 14.4±2.2 | <0.0001 |
| Fat (g/day) | 25.5±14.6 | 27.8±15.0 | 34.0±16.3 | 40.6±17.6 | <0.0001 |
| Fat (%) | 12.9±5.1 | 13.6±5.0 | 15.4±4.8 | 17.1±4.6 | <0.0001 |
| Carbohydrate (g/day) | 315.4±75.1 | 322.5±77.4 | 339.9±82.7 | 355.7±89.4 | <0.0001 |
| Carbohydrate (%) | 74.0±7.1 | 73.2±6.9 | 70.7±6.7 | 68.5±6.4 | <0.0001 |
| Women (n=36 393) | 5 277 | 13 011 | 13 900 | 4 205 |  |
| Person-year | 25 722.4 | 66 000.1 | 69 972.6 | 20 658.9 |  |
| Cases/Total participants (n) | 201/5 277 | 495/13 011 | 489/13 900 | 150/4 205 |  |
| Age (years) | 58.2 ± 7.8 | 56.9 ± 7.6 | 57.2 ± 7.5 | 58.0 ± 7.4 | <0.0001 |
| BMI (kg/m^2^) | 23.5 ± 3.1 | 23.6 ± 3.0 | 23.5 ± 2.9 | 23.4 ± 2.9 | <0.0001 |
| Educational level |  |  |  |  | <0.0001 |
| Middle school or less | 2 117 (40.6) | 4 564 (35.5) | 4 393 (32.0) | 1 331 (32.0) |  |
| High school or college | 2 223 (42.6) | 5 769 (44.8) | 6 355 (46.3) | 1 860 (44.7) |  |
| Undergraduate school or higher | 878 (16.8) | 2 541 (19.7) | 2 978 (21.7) | 967 (23.3) |  |
| Smoking, n (%) |  |  |  |  | 0.0053 |
| Non | 5 101 (97.1) | 12 636 (97.6) | 13 507 (97.6) | 4 069 (97.2) |  |
| Ever | 50 (1.0) | 101 (0.8) | 126 (0.9) | 57 (1.4) |  |
| Current | 102 (1.9) | 208 (1.6) | 200 (1.5) | 60 (1.4) |  |
| Alcohol drinking, n (%) |  |  |  |  | <0.0001 |
| Non | 3 756 (72.4) | 8 777 (69.0) | 9 141 (67.2) | 2 843 (68.8) |  |
| Current | 1 433 (27.6) | 3 941 (31.0) | 4 454 (32.8) | 1 287 (31.2) |  |
| Regular exercise |  |  |  |  | <0.0001 |
| No | 2 755 (52.3) | 6 465 (49.8) | 6 055 (43.7) | 1 610 (38.4) |  |
| Yes | 2 512 (47.7) | 6 506 (50.2) | 7 794 (56.3) | 2 582 (61.6) |  |
| Dietary intake |  |  |  |  |  |
| Total energy intake (kcal/day) | 1 444.9±409.9 | 1 478.5±424.1 | 1 580.2±446.3 | 1 692.2±487.6 | <0.0001 |
| Protein (g/day) | 50.7±18.9 | 52.7±19.8 | 60.0±21.3 | 68.2±24.0 | <0.0001 |
| Protein (%) | 12.9±2.4 | 13.1±2.4 | 13.8±2.5 | 14.5±2.4 | <0.0001 |
| Fat (g/day) | 20.4±11.9 | 23.0±13.2 | 28.6±14.2 | 34.9±15.7 | <0.0001 |
| Fat (%) | 11.5±4.8 | 12.6±5.0 | 14.6±5.0 | 16.6±5.0 | <0.0001 |
| Carbohydrate (g/day) | 292.0±79.0 | 294.7±83.3 | 307.8±85.1 | 322.1±89.6 | <0.0001 |
| Carbohydrate (%) | 75.6±6.7 | 74.2±6.9 | 71.5±7.0 | 68.9±6.9 | <0.0001 |

^1^ Values are presented as the means SD or as n (%). Continuous variables are reported as the mean±SD, while categorical variables are reported as n (%). Total energy intake is adjusted using the residual method.

^2^ p values for categorical and continuous variables are calculated using the chi-square test and general linear regression, respectively.
