# Supplementary material for: Dairy product consumption and type 2 diabetes among Korean adults: a prospective cohort study based on the Health Examinees (HEXA) study
Source: Epidemiol Health. 2022 Feb 4;44:e2022019. doi: 10.4178/epih.e2022019 (PMC9117095; doi:10.4178/epih.e2022019)
Supplement: Supplementary Material 2. — Participant characteristics by yogurt consumption by the HEXA study population [file epih-44-e2022019-suppl2.docx]

Supplementary Material 2. Participant characteristics by yogurt consumption by the HEXA study population

| Yogurt consumption (Serving) | | | | | |
| --- | --- | --- | --- | --- | --- |
| Variables^1^ | Non | ≤2/week | >2-<7/week | ≥1/day | *p*-Value^2^ |
| Men (n=16 895) | 6 276 | 6 826 | 1 882 | 1 911 |  |
| Person-year | 30 670.6 | 33 586.3 | 9 375.6 | 9 623.7 |  |
| Cases/Total participants (n) | 422/6 276 | 404/6 826 | 114/1 882 | 105/1 911 |  |
| Age (years) | 59.6 ± 8.3 | 58.3 ± 8.3 | 58.9 ± 8.4 | 59.9 ± 8.2 | <0.0001 |
| BMI (kg/m^2^) | 24.3 ± 2.7 | 24.3 ± 2.7 | 24.4 ± 2.7 | 24.3 ± 2.8 | 0.6255 |
| Educational level |  |  |  |  | <0.0001 |
| Middle school or less | 1 554 (25.1) | 1 228 (18.2) | 300 (16.1) | 331 (17.5) |  |
| High school or college | 2 582 (41.6) | 2 710 (40.1) | 746 (40.0) | 726 (38.5) |  |
| Undergraduate school or higher | 2 065 (33.3) | 2 820 (41.7) | 819 (43.9) | 831 (44.0) |  |
| Smoking, n (%) |  |  |  |  | <0.0001 |
| Non | 1 699 (27.2) | 2 215 (32.6) | 657 (35.1) | 679 (35.6) |  |
| Ever | 2 657 (42.5) | 2 751 (40.4) | 750 (40.0) | 800 (42.0) |  |
| Current | 1 898 (30.4) | 1 837 (27.0) | 466 (24.9) | 427 (22.4) |  |
| Alcohol drinking, n (%) |  |  |  |  | 0.0003 |
| Non | 1 179 (20.1) | 1 368 (21.4) | 388 (22.4) | 441 (24.8) |  |
| Current | 4 684 (79.9) | 5 028 (78.6) | 1 347 (77.6) | 1 337 (75.2) |  |
| Regular exercise |  |  |  |  | <0.0001 |
| No | 2 777 (44.5) | 2 776 (40.8) | 648 (34.6) | 625 (32.8) |  |
| Yes | 3 471 (55.6) | 4 037 (59.3) | 1 226 (65.4) | 1 281 (67.2) |  |
| Dietary intake |  |  |  |  |  |
| Total energy intake (kcal/day) | 1 678.3±427.1 | 1 732.5±434.7 | 1 805.0±466.7 | 1 789.6±464.3 | <0.0001 |
| Protein (g/day) | 58.7±21.7 | 61.5±21.7 | 70.5±24.6 | 71.9±25.8 | <0.0001 |
| Protein (%) | 13.3±2.5 | 13.4±2.3 | 14.0±2.3 | 13.9±2.4 | <0.0001 |
| Fat (g/day) | 27.6±15.4 | 29.7±15.2 | 35.6±16.9 | 35.5±18.4 | <0.0001 |
| Fat (%) | 13.7±5.3 | 14.3±4.9 | 15.6±4.9 | 15.2±5.0 | <0.0001 |
| Carbohydrate (g/day) | 315.6±76.2 | 327.2±77.9 | 349.2±84.2 | 359.1±87.8 | <0.0001 |
| Carbohydrate (%) | 73.0±7.3 | 72.4±6.7 | 70.4±6.7 | 70.9±6.9 | <0.0001 |
| Women (n=36 393) | 10564 | 15 143 | 5 344 | 5 342 |  |
| Person-year | 51 965.4 | 75 878 | 27 352.3 | 27 158.3 |  |
| Cases/Total participants (n) | 415/10 564 | 549/15 143 | 183/5 344 | 188/5 342 |  |
| Age (years) | 58.0 ± 7.9 | 56.6 ± 7.5 | 57.1 ± 7.2 | 58.3 ±7.2 | <0.0001 |
| BMI (kg/m^2^) | 23.6 ± 3.0 | 23.6 ± 3.0 | 23.4 ± 2.8 | 23.4 ± 2.9 | <0.0001 |
| Educational level |  |  |  |  | <0.0001 |
| Middle school or less | 4 180 (40.1) | 4 934 (32.9) | 1 630 (30.8) | 1 661 (31.5) |  |
| High school or college | 4 475 (42.9) | 6 767 (45.2) | 2 494 (47.1) | 2 471 (46.9) |  |
| Undergraduate school or higher | 1 773 (17.0) | 3 286 (21.9) | 1 168 (22.1) | 1 137 (21.6) |  |
| Smoking, n (%) |  |  |  |  | 0.0002 |
| Non | 10 199 (97.0) | 14 722 (97.7) | 5 214 (98.0) | 5 178 (97.4) |  |
| Ever | 105 (1.0) | 128 (0.9) | 40 (0.8) | 61 (1.2) |  |
| Current | 213 (2.0) | 213 (1.4) | 68 (1.3) | 76 (1.4) |  |
| Alcohol drinking, n (%) |  |  |  |  | <0.0001 |
| Non | 7 327 (70.7) | 9 954 (67.3) | 3 579 (68.3) | 3 657 (69.8) |  |
| Current | 3 038 (29.3) | 4 839 (32.7) | 1 658 (31.7) | 1 580 (30.2) |  |
| Regular exercise |  |  |  |  | <0.0001 |
| No | 5 356 (50.9) | 7 184 (47.6) | 2 223 (41.7) | 2 122 (39.8) |  |
| Yes | 5 172 (49.1) | 7 911 (52.4) | 3 103 (58.3) | 3 208 (60.2) |  |
| Dietary intake |  |  |  |  |  |
| Total energy intake (kcal/day) | 1 501.9±430.3 | 1 535.1±442.5 | 1 568.1±453.8 | 1 581.9±466.1 | <0.0001 |
| Protein (g/day) | 52.5±19.5 | 54.8±20.2 | 62.7±21.7 | 66.5±24.3 | <0.0001 |
| Protein (%) | 13.3±2.5 | 13.4±2.4 | 14.0±2.4 | 14.0±2.5 | <0.0001 |
| Fat (g/day) | 22.9±13.1 | 25.1±13.7 | 30.1±14.9 | 31.6±16.0 | <0.0001 |
| Fat (%) | 12.8±5.3 | 13.5±5.1 | 14.8±5.1 | 14.7±5.0 | <0.0001 |
| Carbohydrate (g/day) | 287.8±79.3 | 296.4±82.5 | 317.5±85.3 | 333.7±90.2 | <0.0001 |
| Carbohydrate (%) | 73.9±7.4 | 73.1±7.0 | 71.2±7.0 | 71.2±7.0 | <0.0001 |

^1^ Values are presented as the mean±SD or as n (%). Continuous variables are reported as the mean±SD, while categorical variables are reported as n (%). Total energy intake is adjusted using the residual method.

^2^ p values for categorical and continuous variables are calculated using the chi-square test and general linear regression, respectively.
