# Supplementary material for: Dairy product consumption and type 2 diabetes among Korean adults: a prospective cohort study based on the Health Examinees (HEXA) study
Source: Epidemiol Health. 2022 Feb 4;44:e2022019. doi: 10.4178/epih.e2022019 (PMC9117095; doi:10.4178/epih.e2022019)
Supplement: Supplementary Material 3. — Participant characteristics by cheese consumption by the HEXA study population [file epih-44-e2022019-suppl3.docx]

Supplementary Material 3. Participant characteristics by cheese consumption by the HEXA study population

| Cheese consumption (serving) | | | | |
| --- | --- | --- | --- | --- |
| Variables^1^ | Non | <1/week | ≥1/week | *p*-Value^2^ |
| Men (n=16 895) | 10 850 | 4 941 | 1 104 |  |
| Person-year | 53 631.6 | 24 167.2 | 5 457.4 |  |
| Cases/Total participants (n) | 715/10 850 | 280/4 941 | 50/1 104 |  |
| Age (years) | 60.0 ± 8.2 | 57.2 ± 8.3 | 58.1 ± 8.6 | <0.0001 |
| BMI (kg/m^2^) | 24.2 ± 2.7 | 24.5 ± 2.7 | 24.5 ± 2.9 | <0.0001 |
| Educational level |  |  |  | <0.0001 |
| Middle school or less | 2 781 (25.9) | 554 (11.3) | 78 (7.1) |  |
| High school or college | 4 560 (42.5) | 1 870 (38.3) | 334 (30.5) |  |
| Undergraduate school or higher | 3 393 (31.6) | 2 460 (50.4) | 682 (62.3) |  |
| Smoking, n (%) |  |  |  | 0.0833 |
| Non | 3 314 (30.6) | 1 574 (32.0) | 362 (32.9) |  |
| Ever | 4 529 (41.9) | 1 969 (40.1) | 460 (41.8) |  |
| Current | 2 978 (27.5) | 1 372 (27.9) | 278 (25.3) |  |
| Alcohol drinking, n (%) |  |  |  | 0.2176 |
| Non | 2 199 (21.8) | 952 (20.5) | 225 (21.8) |  |
| Current | 7 903 (78.2) | 3 687 (79.5) | 806 (78.2) |  |
| Regular exercise |  |  |  | <0.0001 |
| No | 4 617 (42.7) | 1 854 (37.7) | 355 (32.2) |  |
| Yes | 6 203 (57.3) | 3 066 (62.3) | 746 (67.8) |  |
| Dietary intake |  |  |  |  |
| Total energy intake (kcal/day) | 1 677.8±412.4 | 1 780.8±449.6 | 1 968.4±556.0 | <0.0001 |
| Protein (g/day) | 60.0±21.8 | 65.8±23.2 | 75.0±27.5 | <0.0001 |
| Protein (%) | 13.3±2.4 | 13.7±2.3 | 14.4±2.4 | <0.0001 |
| Fat (g/day) | 28.1±15.3 | 32.9±16.1 | 39.8±19.3 | <0.0001 |
| Fat (%) | 13.7±5.0 | 15.1±4.9 | 17.0±5.1 | <0.0001 |
| Carbohydrate (g/day) | 323.3±77.5 | 336.7±82.6 | 350.0±92.8 | <0.0001 |
| Carbohydrate (%) | 73.0±7.0 | 71.2±6.8 | 68.6±7.0 | <0.0001 |
| Women (n=36 393) | 19 305 | 12 182 | 4 906 |  |
| Person-year | 97 340.2 | 60 771 | 24242.8 |  |
| Cases/Total participants (n) | 787/19 305 | 397/12 182 | 151/4 906 |  |
| Age (years) | 58.3 ± 7.6 | 55.9 ± 7.3 | 56.9 ± 7.3 | <0.0001 |
| BMI (kg/m^2^) | 23.7 ± 3.0 | 23.4 ± 2.9 | 23.1 ± 2.9 | <0.0001 |
| Educational level |  |  |  | <0.0001 |
| Middle school or less | 8 544 (44.8) | 2 938 (24.4) | 923 (19.0) |  |
| High school or college | 8 066 (42.3) | 5 848 (48.6) | 2 293 (47.2) |  |
| Undergraduate school or higher | 2 471 (13.0) | 3 250 (27.0) | 1 643 (33.8) |  |
| Smoking, n (%) |  |  |  | 0.0172 |
| Non | 18 770 (97.6) | 11 796 (97.5) | 4 747 (97.1) |  |
| Ever | 151 (0.8) | 120 (1.0) | 63 (1.3) |  |
| Current | 308 (1.6) | 185 (1.5) | 77 (1.6) |  |
| Alcohol drinking, n (%) |  |  |  | <0.0001 |
| Non | 13 486 (71.2) | 7 877 (66.3) | 3 154 (65.8) |  |
| Current | 151 (0.8) | 120 (1.0) | 63 (1.3) |  |
| Regular exercise |  |  |  | <0.0001 |
| No | 9 522 (49.5) | 5 476 (45.1) | 1 887 (38.6) |  |
| Yes | 9 735 (50.6) | 6 659 (54.9) | 3 000 (61.4) |  |
| Dietary intake |  |  |  |  |
| Total energy intake (kcal/day) | 1 486.4±416.1 | 1 555.6±451.6 | 1 691.2±497.4 | <0.0001 |
| Protein (g/day) | 53.5±19.8 | 58.7±21.8 | 66.3±24.0 | <0.0001 |
| Protein (%) | 13.2±2.4 | 13.7±2.4 | 14.3±2.5 | <0.0001 |
| Fat (g/day) | 23.1±12.9 | 27.8±14.4 | 33.7±16.4 | <0.0001 |
| Fat (%) | 12.6±5.0 | 14.4±5.0 | 16.2±5.3 | <0.0001 |
| Carbohydrate (g/day) | 296.8±81.5 | 305.3±86.2 | 317.6±90.8 | <0.0001 |
| Carbohydrate (%) | 74.1±6.9 | 71.9±7.0 | 69.4±7.3 | <0.0001 |

^1^ Values are presented as the means±SD or as n (%). Continuous variables are reported as the mean (standard deviation) values, while categorical variables are reported as n (%). Total energy intake is adjusted using the residual method.

^2^ p values for categorical and continuous variables are calculated using the chi-square test and general linear regression, respectively.
